# Coarse-Graining Waters: Unveiling The Effective Hydrophilicity/Hydrophobicity of Individual Protein Atoms and The Roles of Waters’ Hydrogens

**DOI:** 10.1021/acs.jctc.3c00700

**Published:** 2023-10-02

**Authors:** Hyuntae Na, Guang Song

**Affiliations:** † Department of Computer Science, Penn State Harrisburg, Middletown, Pennsylvania 17057, United States; ‡ Department of Mathematics and Computer Science, Westmont College, Santa Barbara, California 93108, United States

## Abstract

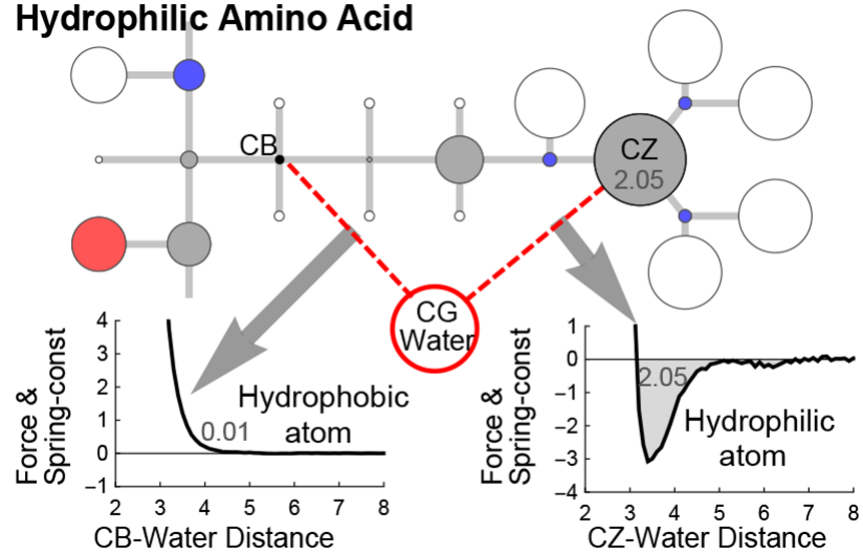

There have been many coarse-graining methods developed that aim to reduce the sizes of simulated systems and their computational costs. In this work, we develop a new coarse-graining method, called coarse-graining-delta (or δ-CG in short), that reduces the degrees of freedom of the potential energy surface by coarse-graining relative locations of atoms from their unit centers. Our method extends and generalizes the methods used in the coarse-grained normal mode analysis and enables us to study the roles of the individual removed atoms in a system, which have been difficult to study in molecular dynamics simulations. By applying δ-CG to coarse-grain three-point water molecules into single-point solvent particles, we successfully identify the effective hydrophilicity and hydrophobicity of all the individual protein atom types, which collectively correlate well with the known hydrophilic, hydrophobic, and amphipathic characteristics of amino acids. Moreover, our investigation shows that water’s hydrogens have two roles in interacting with protein atoms. First, water molecules adjust their poses around different amino acids and their atoms, and the statistical preferences of the hydrogen poses near the atoms determine the effective hydrophilicity and hydrophobicity of the atoms, which have not been successfully addressed before. Second, the collective dynamics of the hydrogens assist the water molecules in escaping from the potential energy wells of the hydrophilic atoms. Our method also shows that coarse-graining a system mathematically leads to breaking antisymmetry of the nonbonded interactions; as a result, two interacting coarse-grained units exert different forces on each other. Our study indicates that the accuracy of coarse-grained force fields, such as the MARTINI force field and the UNRES force field, can be improved in two ways: (i) refining their potential energy functions and coefficients by analyzing the coarse-grained potential energy surface using δ-CG, and (ii) introducing non-antisymmetric interactions.

## 1 Introduction

Studying the dynamics of biomolecular systems requires extensive computational resources and takes a significant amount of computational time in many cases. This is because biomolecular systems often contain hundreds of thousands of atoms or more and simulating their dynamics requires handling their interactions. To reduce the computational cost, coarse-graining approaches or coarse-grained models have been developed. The coarse-graining approaches are often used to reduce both the degrees of freedom of biomolecules, such as proteins, lipids, and nucleic acids, and those of their surrounding environment, such as water molecules. For example, the MARTINI coarse-grained force field^1−3^ is often used with its coarse-grained water models.^4,5^ The coarse-grained force fields and the coarse-grained water models are generally optimized by fitting their coefficients to the potential of mean force (PMF)^6^ or by using the force-matching (FM) method^7^ at the coarse-grained level.

Coarse-grained water models can be categorized into three types: one-site, two-site, and cluster models. The one-site models represent each water molecule using one point, which is generally the water’s center of mass or its oxygen location.^8−11^ Their common issue is that it is unclear how to handle electrostatic interactions because of the electrostatic neutrality of each water molecule: its net charge is zero. Therefore, the models are usually designed to describe interactions between different water molecules^9^ rather than interactions between water molecules and biomolecules, such as proteins. The two-site models represent each water molecule using two points.^8,12−14^ The additional point is used to describe the point dipole of the water molecule to capture the electrostatic interactions with biomolecules. However, the models may not provide enough computational speedups compared with the one-site models and the cluster models. The cluster models represent a group or cluster of water molecules using 2–4 points.^4,15−20^ Using multiple points enables capturing the electrostatic interactions, and using the 2–4 points per cluster provides computational speedups. The cluster models are widely used with coarse-grained force fields, such as the MARTINI force field,^1−3^ to efficiently simulate the molecular dynamics of biomolecules in solvents.

One may consider that the cluster models are sufficient to describe interactions between the biomolecules and the water molecules. However, there are certain benefits of using one-site models if they are capable of describing electrostatic interactions with the biomolecules. For example, using the one-site models can describe how the biomolecules behave or function through the complex water–water interaction network, especially in the hydration shell. More fundamentally, modeling an individual water molecule as an independent particle is more appropriate rather than requiring the water molecules to be bound up with a cluster unit. Using the cluster models is difficult in simulating the dispersion of water molecules and the situation where a trapped water molecule (or a thin layer of water molecules) mediates interactions between two biomolecules at their interfaces.

Coarse-graining approaches have also been applied in normal mode analysis (NMA) and elastic network models (ENMs) that are a subset of normal mode analysis. Methods of coarse-graining a system in NMA and ENMs can be grouped into three categories: extracting dynamics of sparsely distributed atoms in the system,^21−23^ considering subunits of the system as rigid units,^24−28^ and reducing the dimension of the system’s dynamics considering its symmetricity^29^ or the resonance between its building units.^30,31^ The methods in the first category have two interesting properties. First, the methods reduce the size of the Hessian matrix of the system by projecting or embedding information on nonkeeping atoms (or typically non-C^α^ atoms in proteins) into keeping atoms (or C^α^ atoms). Second, the methods maintain the keeping atoms’ dynamics to be the same as those in the original Hessian matrix, in terms of the mean-square fluctuation and the cross-correlation that make it easy to understand the dynamics of the coarse-grained system. The methods can be considered as reducing the degrees of freedom of the system’s conformational space by assuming that nonkeeping atoms move in the direction of reducing the potential energy change of the system.^22,32^

Coarse-graining a system using the above assumption used in the NMA-based methods is more sound than coarse-graining the system by fitting to the potential of mean force (PMF) or using the force-matching (FM) method with molecular dynamics (MD) trajectories. However, there is a constraint in the NMA-based methods: the system must be in an equilibrium where the net force of each atom is zero for all atoms. To resolve this constraint, we have proposed another coarse-graining method that extends and generalizes the NMA-based methods by eliminating the equilibrium constraint.^32^

In this work, we develop a new coarse-graining method that further extends our previous work,^32^ and then apply the method to coarse-grain water molecules to one-site solvent particles while maintaining the capability of describing electrostatic interactions with proteins. We call it the coarse-graining-delta (or δ-CG), which will be explained in the Methods section. Our method, instead of targeting maintaining the dynamics of keeping atoms, considers each coarse-grained unit (a water molecule in this work) as a whole unit (a solvent particle) and coarse-grains the relative displacements of its elements (namely, the water’s hydrogens and oxygen) using the above assumption. Additionally, our method makes it possible to observe how each element contributes to the potential energy surface formed by the coarse-grained unit. Even though our method is applied to coarse-grain water molecules in this work, it can be applied to coarse-grain any units of systems. We demonstrate how our method can be used to (i) coarse-grain water molecules represented by the TIP3P water model, (ii) extract pairwise forces and spring constants between the coarse-grained solvent particle and protein’s atoms, and (iii) determine the roles of water’s hydrogens in interacting with the protein’s atoms. We additionally show that the hydrophilicity and hydrophobicity of the protein’s atoms can be revealed using our method, which accurately reproduces the hydrophilic, hydrophobic, and amphipathic characteristics of various amino acids.

## 2 Methods

### 2.1 The Coarse-Graining-Delta Method

Here, we describe the details of the proposed coarse-graining-delta method that reduces the degrees of freedom of the potential energy surface of a system by coarse-graining each three-point water molecule as a single-point solvent particle. Given a system composed of a protein (or proteins) and water molecules, the potential energy *V* of the system can be approximated by the second-order Taylor expansion, as follows:^32^
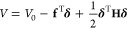
1where T represents the vector (or matrix) transpose, **δ** is a vector whose values are the displacements of atoms from the given conformation, and *V*_0_, **f**, and **H** are the potential energy, the force vector, and the Hessian matrix of the system’s potential energy at the given conformation, respectively. Note that *V* represents the potential energy surface of the system of variable **δ**. The displacement **δ** can be split into the displacement of protein’s atoms **δ**_*p*_, waters’ oxygens **δ**_*o*_, waters’ first-hydrogens **δ**_1_, and second-hydrogens **δ**_2_. We can rewrite **δ** as follows:
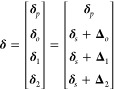
2where **δ**_*s*_ is the displacements of the water centers, and **Δ**_*o*_, **Δ**_1_, and **Δ**_2_ are the relative displacements of the oxygens, first- and second-hydrogens from their corresponding water centers, respectively. To simplify notations, we use the term “solvent particle” to represent a single-point coarse-grained water molecule. Additionally, we will use the terms “atom”, “oxygen” and “hydrogen” to represent a protein’s atom, a water’s oxygen, and a water’s hydrogen, respectively. Note that subscripts *p*, *s*, *o*, 1, and 2 represent the groups of protein’s atoms, solvent particles, oxygens, and first- and second-hydrogens, respectively.

Let the location of an oxygen represent the location of its corresponding solvent particle or water center: **δ**_*s*_ = **δ**_*o*_. This has a benefit in that, when coarse-graining electrostatic and van der Waals interactions between an atom and a water molecule, its atom–oxygen interaction can be simply determined from the distance between the atom and its corresponding solvent particle. We rewrite eq 1 by reorganizing **δ** into **δ̃**, as follows:
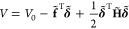
3where
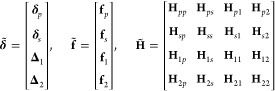
4In the above, **f**_*p*_ is the subvector of the force vector **f** for atoms (or protein’s atoms), and **H**_*pp*_ is the submatrix of the Hessian matrix **H** for interactions between atoms. In a similar manner, **f**_1_ and **f**_2_ are the subvectors for first- and second-hydrogens, respectively; **H**_*p*1_, **H**_*p*2_, **H**_1*p*_, and **H**_2*p*_ are the submatrices for interactions between atoms and first/second-hydrogens; and **H**_11_, **H**_12_, **H**_21_, and **H**_22_ are the submatrices for interaction between first-hydrogens and second-hydrogens. The subvectors and the submatrices involving solvent particles *s* are written using summations of those involving oxygens and hydrogens, as follows:
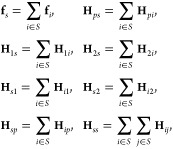
5where *S* = {*o*, 1, 2}. To simplify the notation, denote by **δ**_*ps*_ the vector concatenating **δ**_*p*_ and **δ**_*s*_, and by **H**_*ps*,12_ the matrix concatenating **H**_*p*1_, **H**_*p*2_, **H**_*s*1_, and **H**_*s*2_. In a similar manner, **Δ**_12_, **f**_*ps*_, **f**_12_, **H**_*ps,ps*_, **H**_12,*ps*_ and **H**_12,12_ are denoted.

In ref (32), we have developed a method that coarse-grains the potential energy surface *V* of a non-minimized system by embedding the information on nonkeeping atoms (or atoms to be removed) into the potential energy surface determined by keeping atoms (or atoms to be kept). We utilized our previous method to coarse-grain the system by removing the relative hydrogen displacement terms **Δ**_1_ and **Δ**_2_ in *V*. The following describes how to coarse-grain the system, which is similar to our previous work.^32^ We remove the relative hydrogen displacements (or simply hydrogens) **Δ**_1_ and **Δ**_2_ in eqs 3 and 4 by embedding their information into the potential energy surface *V* determined by the atoms **δ**_*p*_ and the solvent particles **δ**_*s*_. To this end, we assume the following condition. While the atoms **δ**_*p*_ and the solvent particles **δ**_*s*_ can freely move toward any direction, the motion direction of the hydrogens **Δ**_1_ and **Δ**_2_ are constrained to maintain the potential energy of the system in the mass-weighted coordinates. The condition implies that **δ**_*p*_ and **δ**_*s*_ are random variables, and **Δ**_1_ and **Δ**_2_ are constrained variables. The condition is derived from the assumption that there is no energy exchange between the potential and kinetic energies in the system. The condition yields the partial derivatives of the potential energy *V* in eq 4 with respect to **Δ**_1_ and **Δ**_2_ (or simply **Δ**_12_) in the mass-weighted coordinates **Δ̃**_12_ = **M**_12_^–1/2^**Δ**_12_ be zero:
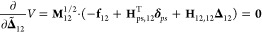
6where **M**_12_ is the masses of waters’ hydrogens in a matrix form, and **0** is a vector whose all values are zero. We coarse-grain the potential energy surface *V* by plugging **Δ**_12_ in eq 6 into eq 3, as follows:
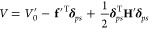
7where
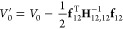
8
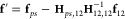
9
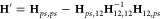
10In the above, *V*_0_^′^, **f**′, and **H**′ are the potential energy, the force vector, and the Hessian matrix of the coarse-grained system’s potential energy at the given conformation, respectively. They determine the shape of the potential energy surface *V* of the system using only the locations of atoms **δ**_*p*_ and solvent particles **δ**_*s*_. Note that the first summands *V*_0_, **f**_*ps*_, and **H**_*ps,ps*_ in eqs 8–10 are *directly* determined from the locations of the atoms, the oxygens, and the hydrogens at the given conformation. The second summands are *indirectly* determined by the collective dynamics of the hydrogens **Δ**_12_ that preserve the potential energy of the system. In the rest of this paper, we will refer to the collective dynamics as *the collective hydrogen dynamics*.

We call this method the coarse-graining-delta (or δ-CG) since it removes the relative displacements of hydrogens from their water centers **Δ**_1_ and **Δ**_2_ instead of their actual displacements **δ**_1_ and **δ**_2_. This method generalizes and extends the coarse-graining approaches used in the normal mode analysis (NMA): (i) the conformation of the system does not need to be in a local energy minimum, and (ii) the coarse-graining equations in eqs 7–10 become equal to the coarse-graining equations used in NMA when the force is zero (**f** = **0**) and the water center location is set to be the origin (**δ**_*s*_ = **0**) in eq 2.

### 2.2 Extracting Pairwise Force

One of our goals in this work is to examine individual interactions between atoms and solvent particles. Note that a force **f**_*i*_ of an atom (or oxygen or hydrogen) *i* in eq 1 is calculated by summing all pairwise forces **f**_*i*←*j*_ exerted on *i* by other atoms (or oxygens or hydrogens) *j*: **f**_*i*_ = ∑_*j*≠*i*_**f**_*i*←*j*_. To observe the individual interactions in a coarse-grained system, we decomposed force **f**′ in eq 9 into pairwise forces.

Let **F** be the 3*n* × *n* pairwise force matrix, which can be partitioned into *n* × *n* blocks whose (*i*, *j*) entry is a 3 × 1 vector representing the pairwise force exerted on an atom *i* by another atom *j* in 3-D. The value *n* is the total number of atoms including protein’s and water molecules’ atoms. The 3*n* × 1 force vector **f** in eq 1 can be rewritten using **F**, as follows:
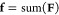
11In the above, sum(**A**) is the function summing all column vectors of a matrix **A** and is defined as follows:
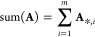
12where *m* is the number of columns in **A**, and A_*,i_ is the *i*-th column vector of **A**. In a similar manner, force vector **f**′ in eq 9 can be rewritten. Let *n*_*p*_ and *n*_*s*_ be the number of atoms and that of solvent particles (composed of oxygens and first- and second-hydrogens), respectively: *n* = *n*_*p*_ + 3*n*_*s*_. Denote by **F**_*s*←*p*_ the 3*n*_*s*_ × *n*_*p*_ matrix that represents pairwise forces exerted on the *n*_*s*_ solvent particles by the *n*_*p*_ atoms, which can be calculated as follows:
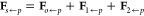
13In the above, **F**_*o*←*p*_ (and **F**_1←*p*_ and **F**_2←*p*_) is the 3*n*_*s*_ × *n*_*p*_ submatrix of **F**, representing pairwise forces exerted on the *n*_*s*_ oxygens (and first- and second-hydrogens) by the *n*_*p*_ atoms. In a similar manner, the pairwise force matrices **F**_*p*←*s*_, **F**_*s*←*s*_, **F**_1←*s*_, and **F**_2←*s*_ are calculated by summing submatrices of **F**. Similarly, **F**_*p*←*p*_ is the 3*n*_*p*_ × *n*_*p*_ submatrix of **F**, representing pairwise forces between the *n*_*p*_ atoms. Now, eq 9 can be rewritten as follows:
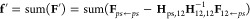
14where
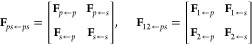
15The (*i*, *j*) entry of **F**′ can be written, as follows:
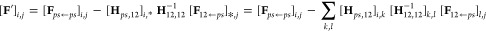
16where [**F**]_*a,b*_ is a 3 × 1 vector that is the (*a*, *b*) entry of a pairwise force matrix **F**, [**H**]_*a,b*_ is a 3 × 3 matrix that is the (*a*, *b*) entry of a Hessian matrix **H**, and * in subscript represents the whole entries in the row or column of the matrix. In the above, the first summation indicates the force *directly* exerted on *i* by *j*. The second summation indicates the force *indirectly* exerted on *i* by *j* through the collective hydrogen dynamics. It can also be understood as the force indirectly exerted on *i* by *j* through the chain of (i) the force from *j* to a hydrogen *l*, (ii) the mediation conveyed by two hydrogens *l* and *k* through their collective dynamics in **H**_12,12_^–1^, and (iii) the interaction between *k* and *i*.

#### Coarse-Graining Breaks the Antisymmetry of Forces in Nonbonded Interactions

Note that all pairwise forces between nonbonded atoms are antisymmetric (or skew-symmetric): a force **f**_*a*←*b*_ exerted on an atom *a* by another atom *b* is opposite to a force **f**_*b*←*a*_ exerted on *b* by *a*: **f**_*a*←*b*_ = −**f**_*b*←*a*_. However, eq 16 shows that coarse-graining a system breaks the antisymmetric property between pairwise forces. In the equation, the first summand matrix **F**_*ps*←*ps*_ is antisymmetric: [**F**_*ps*←*ps*_]_*i*,*j*_ = −[**F**_*ps*←*ps*_]_*j*,*i*_. This indicates that the *directly* exerted pairwise forces in nonbonded interactions between atoms and coarse-grained units keeps the antisymmetry. However, the second summand matrix is not antisymmetric both mathematically and in terms of its values: [**H**_*ps*,12_**H**_12,12_^–1^**F**_12←*ps*_]_*i*,*j*_ ≠ −[**H**_*ps*,12_**H**_12,12_^–1^**F**_12←*ps*_]_*j*,*i*_. This indicates that the *indirectly* exerted pairwise forces in the nonbonded interactions through the collective hydrogen dynamics lose the antisymmetry.

This indicates that *coarse-graining a system mathematically leads to breaking the antisymmetry of pairwise forces in the nonbonded interactions*. Therefore, any nonbonded interacting pairs of atoms and/or coarse-grained units exert different forces on each other. This implies that, when describing the dynamics of coarse-grained systems using forces, pairwise forces in nonbonded interactions between the atoms and the coarse-grained units need to be determined from different formulas or coefficients to improve the accuracy of describing the dynamics theoretically. In other words, introducing the non-antisymmetric interactions into the coarse-grained force fields, such as the MARTINI force field^2−5^ and the UNRES force field,^33,34^ may improve the accuracy of the dynamics when running molecular dynamics simulations.

### 2.3 Restructuring Pairwise Forces to Be Antisymmetric

We restructured the pairwise force **F**′ in eq 16 to be antisymmetric. This is because using antisymmetric pairwise forces is convenient to analyze interactions between atoms and solvent particles. Return to force vector **f**′ in eqs 7 and 9. The second-summand force term in the potential energy surface *V* in eq 7 can be rewritten using the pairwise forces in eq 16, as follows:
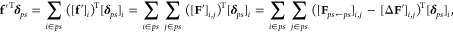
17where [**a**]_*i*_ is a 3 × 1 subvector of a vector **a** for an atom *i*, and
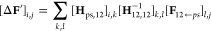
18In the above, [Δ**F**′]_*i,j*_ is equal to the second summand in eq 16, which indicates the force *indirectly* exerted on *i* by *j* through the collective hydrogen dynamics. Note that Δ**F**′ and **F**′ are not antisymmetric. We rearrange the components of **F**′ to make it antisymmetric without altering the potential energy surface *V*. Equation 17 can be rewritten using the antisymmetric pairwise force **F**″, as follows:
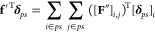
19where
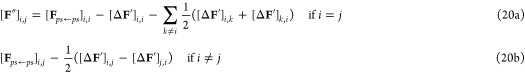
20Note that [Δ**F**′]_*i,j*_ and – [Δ**F**′]_*j,i*_ have a similar meaning even though they are different both mathematically and in terms of their values: −[Δ**F**′]_*j,i*_ is the reversed direction of the force exerted on *j* by *i* through the collective hydrogen dynamics. Therefore, the second summand ^1^/_2_([Δ**F**′]_*i,j*_ – [Δ**F**′]_*j,i*_) in eq 20b redefines the force [Δ**F**′]_*i,j*_ exerted on *i* by *j* in eq 18 using the arithmetic average. The meaning of the second summand ^1^/_2_([Δ**F**′]_*i,k*_ + [Δ**F**′]_*k,i*_) in eq 20a cancels out. As a result, **F**″ approximates **F**′ without altering the potential energy surface *V* and becomes antisymmetric: [**F**″]_*i,j*_ = −[**F**″]_*j,i*_.

### 2.4 Decomposing the Pairwise Force Vector and the Hessian Matrix into Detailed Direct and Indirect Interaction Components

One of our goals in this work is to investigate the details of how atoms and solvent particles interact. We decompose the pairwise force vector and the Hessian matrix describing the interaction between an atom *a* and a solvent particle *b* into distinct components to determine their contributions.

The (*a*, *b*) entry of the Hessian matrix **H**′ in eq 10 is a 3 × 3 matrix describing the tensor spring between *a* and *b*. The entry [**H**′]_*a,b*_ is composed of two summands:
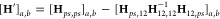
21Recall that its first summand represents the direct interaction between *a* and *b* and is composed of electrostatic and van der Waals interactions between their atoms; its second summand represents their indirect interaction mediated by the collective hydrogen dynamics. The direct interaction can be further decomposed, and the above equation can be rewritten as follows:
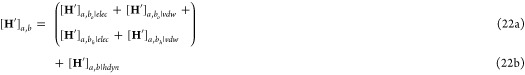
22where *b*_*o*_ and *b*_*h*_ stand for the oxygen and the two hydrogens of the solvent particle *b*, respectively, *elec* and *vdw* stand for the electrostatic and the van der Waals interactions, respectively, and *hdyn* stands for the interaction mediated by the collective hydrogen dynamics. The first four summands in eq 22a compose the direct interaction. The last sum in eq 22b composes the indirect interaction: [**H**′]_*a,b*|*hdyn*_ = −[**H**_*ps*,12_**H**_12,12_^–1^**H**_12,*ps*_]_*a,b*_.

The (*a*, *b*) entry of the pairwise force matrix **F**″ in eq 20b is a 3 × 1 vector describing the force exerted on *a* by *b* after making it antisymmetric. In a similar manner, entry [**F**″]_*a,b*_ can be decomposed and rewritten as follows:
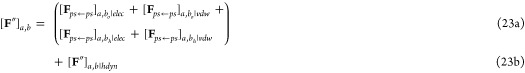
23In the above, the first four summands compose the direct interaction, and they are decomposed from the first summand [**F**_*ps*←*ps*_]_*a,b*_ in eq 20b. The last summand composes the indirect interaction, and it is equal to the second summand in eq 20b: [**F**″]_*a,b*|*hdyn*_ = −^1^/_2_([Δ**F**′]_*a,b*_ – [Δ**F**′]_*b,a*_).

### 2.5 The Effective Pairwise Potential

Equations 7–10 make it possible to observe how proteins and solvent particles interact in the potential energy surface at the mixed-grained level. Equation 20 decomposes the coarse-grained force in eq 9 into the antisymmetric pairwise forces. Here, we develop a foundation of the pairwise potentials that can describe the pairwise interactions between an atom and a solvent particle or between two solvent particles.

The potential energy surface *V* of a system can be approximated by the second-order Taylor expansion, as explained in eq 1. Many-body interactions can be decomposed into a list of two-body interactions, even though each two-body interaction should be written using both vectors and matrices rather than scalars. Assume that all spring constant matrices for any two interacting atoms/particles *i* and *j* are symmetric and all pairwise forces are antisymmetric: [**H**]_*i,j*_ = [**H**]_*j,i*_ and [**F**]_*i,j*_ = −[**F**]_*j,i*_. This is especially true for two-body and pair potentials, such as potentials for the bond stretching and the nonbonded interactions. Using the assumption, eq 1 or 3 can be rewritten as a sum of pair potentials, where each of which is approximated by the second-order Taylor expansion, as follows:
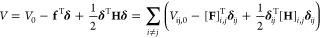
24where **δ**_*ij*_ = [**δ**]_*i*_ – [**δ**]_*j*_, and *V*_*ij*,0_ is the potential energy between *i* and *j* given their locations. In the above, [**F**]_*i,j*_ is a 3 × 1 vector representing the force exerted on *i* by *j*, and [**H**]_*i,j*_ is a 3 × 3 matrix representing the tensor spring between *i* and *j*. In our course-graining problem, [**F**]_*i,j*_ corresponds to [**F**″]_*i,j*_ in eq 23, and [**H**]_*i,j*_ corresponds to [**H**′]_*i,j*_ in eq 22.

Consider that the interaction between *i* and *j* can be approximated by using a linear spring or a pair potential. The potential energy surface *V* can be further simplified using the distance change *r*_*ij*_ between *i* and *j*, as follows:
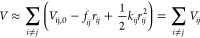
25where
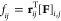
26
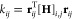
27where **r**_*ij*_ is a unit vector showing the direction . In the above, *V*_*ij*_ represents the pairwise potential energy surface between *i* and *j*. The terms *f*_*ij*_ and *k*_*ij*_ are the force [**F**]_*i,j*_ and the spring constant [**H**]_*i,j*_ in eq 24 projected on the direction **r**_*ij*_, respectively. Let us call *V*_*ij*_, *f*_*ij*_, and *k*_*ij*_ the effective pairwise potentials, the effective pairwise force, and the effective spring constant, respectively. It is valid to use the effective pairwise potentials *V*_*ij*_ to simplify the potential energy surface *V* in eq 24, especially when observing the interactions between nonbonded atoms/particles.

## 3 Results and Discussion

In this section, we demonstrate that our proposed approach is able to (i) capture the details of the interactions between protein atoms and solvent particles using the effective pairwise forces and the effective spring constants, (ii) identify the atoms’ hydrophilicity and hydrophobicity, and (iii) determine the roles of water’s hydrogens in atom-solvent interactions.

### 3.1 Experiment Setup

The following describes how we collect the data set containing the effective pairwise forces and the effective spring constants. First of all, we run a molecular dynamics (MD) simulation to sample MD conformations that capture the protein–solvent interactions at the atomic level. We build a system with a wild-type sperm whale myoglobin (pdb-id: 1A6G) in a water box by adding missing hydrogens in its X-ray structure, removing the carbon monoxide (CO) and the sulfate ions (SO_4_), and immersing it in a water box whose size is about 100 × 100 × 100 Å^3^. The system contains about 2,500 atoms of the myoglobin and about 96,800 atoms of water molecules. The carbon monoxide ligand and the sulfate ions in 1A6G are not included in the system since our study does not target the interactions between the protein and the ligand and the effects of the ions on the system. After minimizing the system, we run an MD simulation for 10 ns. The MD simulation is performed using the Tinker Molecular Modeling program,^35^ the CHARMM22 (Chemistry at Harvard Macromolecular Mechanics) empirical force field,^36^ the TIP3P water model,^37^ the particle mesh Ewald (PME) method, and the condition of 298 K temperature and 1.0 atm pressure. To reduce its computational cost, we ignore nonbonded interactions beyond a 12 Å cutoff distance by gradually reducing the nonbonded potential energy from its standard value at 7.8 Å to zero at the cutoff distance (truncation and shifting). From the 10 ns MD simulation, we collect 951 conformations from its MD trajectory every 10 ps after 0.5 ns, to collect the MD conformations after the MD simulation reached the equilibrium.

For each MD conformation, we subsample it by taking the myoglobin and a 12 Å thick solvent layer, a subset of water molecules whose distances from the protein are less than or equal to 12 Å. The distance between a water molecule and the protein is measured by checking the distance between the center of the water’s oxygen and that of the closest atom of the protein. The subsampled system has about 2,500 atoms of the protein and about 11,000 atoms of water molecules. Our previous study shows that using a 12 Å thick solvent layer is an economical choice: it saves a significant amount of computational time yet keeps around 95% accuracy of protein’s dynamics at the atomic level.^32^

Given a subsampled system, we collect the effective pairwise forces and the effective spring constants of interactions between protein atoms and solvent particles (or simply atom–solvent interactions). Additionally, we also collect those between solvent particles (solvent–solvent interactions). To this end, we first determine the potential energy *V*_0_, the force vector **f**, the Hessian matrix **H**, and the pairwise forces matrix **F** of the potential energy at the conformation of the subsampled system. To avoid potential artifacts introduced by the truncation and shifting, when calculating the electrostatic and van der Waals potentials in this step, we do not make the potentials zero even though their distances are larger than the 12 Å cutoff distance. We then coarse-grain the solvent particles and determine the potential energy *V*_0_^′^, the force vector **f**′ and the Hessian matrix **H**′, representing the potential energy surface of the coarse-grained system, using the proposed coarse-graining-delta method in eqs 7–10. At the same time, we determine the pairwise force matrix **F**′ in eq 16 and its antisymmetric pairwise force matrix **F**″ in eq 20. From **F**″, **H**′, and the conformation, we collect the distance *r*_*ij*_, the effective pairwise forces *f*_*ij*_ in eq 26 and the effective spring constants *k*_*ij*_ in eq 27 for all pairs of a protein atom *i* and a coarse-grained solvent particle *j*. To observe the influence of the viscous behavior of solvent particles on the dynamics of protein atoms, we collect the motion correlations *c*_*ij*_ from **H**′.^21^ Additionally, we collect those of all pairs of two coarse-grained solvent particles *i* and *j*. We selectively keep [**F**″]_*i,j*_, [**H**′]_*i,j*_, *f*_*ij*_, *k*_*ij*_, *r*_*ij*_, and *c*_*ij*_ only when *r*_*ij*_ ≤ 12 Å to save data storage. Each subsampled system has an average of around 289 (and 291) thousand atom–solvent (and solvent–solvent) interactions when their distances are within 12 Å, out of a total of about 14 (and 62) million interactions.

We repeat this process for all 951 MD conformations. In total, we collect around 275 (and 276) million atom–solvent (and solvent–solvent) interactions. The collected [**F**″]_*i,j*_, [**H**′]_*i,j*_, *f*_*ij*_, *k*_*ij*_, *r*_*ij*_, and *c*_*ij*_ of all interacting pairs become our data set. We group the protein atoms into 71 subtypes of carbons, hydrogens, nitrogens, oxygens, and sulfurs considering their partial charges and van der Waals coefficients and their covalently bonded neighbors, based on the atom types listed in the Tinker Molecular Modeling program. The data set is used to study how protein atoms and amino acids interact with solvent particles and how solvent particles interact with each other.

#### Interaction Components

**Figure 1:**
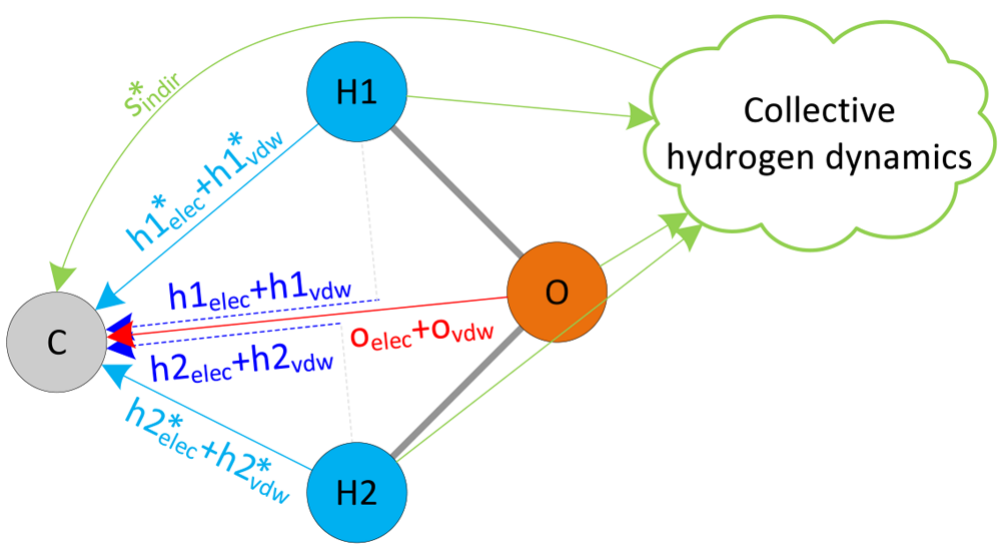
An illustration of the atom-solvent interaction components. Interactions with * in its superscript represent the interactions in 3-D. Interactions without * represent the interactions projected on the  line. The combination of h1_*elec*_ and h2_*elec*_ (and h1_*vdw*_ and h2_*vdw*_) becomes *h*_*elec*_ (and *h*_*vdw*_), i.e., *h*_*elec*_ = *h*1_*elec*_ + *h*2_*elec*_ and *h*_*vdw*_ = *h*1_*vdw*_ + *h*2_*vdw*_.

In this study, we investigate how different atom types of proteins interact with solvent particles by evaluating the contributions of the interaction components. We observe the interaction between an atom *a* and a solvent particle *b* using the effective pairwise force *f*_*ab*_ in eq 26 and the effective spring constant *k*_*ab*_ in eq 27. In our experiments, *f*_*ab*_ and *k*_*ab*_ are determined from [**F**″]_*a,b*_ and [**H**′]_*a,b*_ that are the (*a*, *b*) entries of the antisymmetric pairwise force matrix **F**″ in eq 20 and the Hessian matrix **H**′ in eq 10, respectively. Recall that [**F**″]_*a,b*_ is composed of five summands, as shown in eq 23. For simplicity, we sequentially denote the five summands by *o*_*elec*_, *o*_*vdw*_, *h*_*elec*_, *h*_*vdw*_, and *s*_*indir*_, based on their interaction types. The first four summands in eq 23a represent the direct interaction components: *o*_*elec*_, *o*_*vdw*_, *h*_*elec*_, and *h*_*vdw*_, where *elec* (and *vdw*) stands for electrostatic (and van der Waals) interaction, and *o* (and *h*) stands for the interaction between the atom *a* and the oxygen (and two hydrogens) of the solvent *b*. The total *direct* interaction component is denoted by *s*_*dir*_: *s*_*dir*_ = *o*_*elec*_ + *o*_*vdw*_ + *h*_*elec*_ + *h*_*vdw*_. The fifth summand in eq 23b with *hdyn* in its subscript represents the *indirect* interaction component *s*_*indir*_ that is influenced by the collective hydrogen dynamics. In a similar manner, [**H**′]_*a,b*_ in eq 22 is also composed of the same types of five interaction components: *o*_*elec*_, *o*_*vdw*_, *h*_*elec*_, *h*_*vdw*_, and *s*_*indir*_. We analyze the detailed contributions of the five components in atom–solvent interactions. Figure 1 illustrates the five components of an atom–solvent interaction.

### 3.2 Effective Hydrophilicity and Hydrophobicity of Atoms

**Figure 2:**
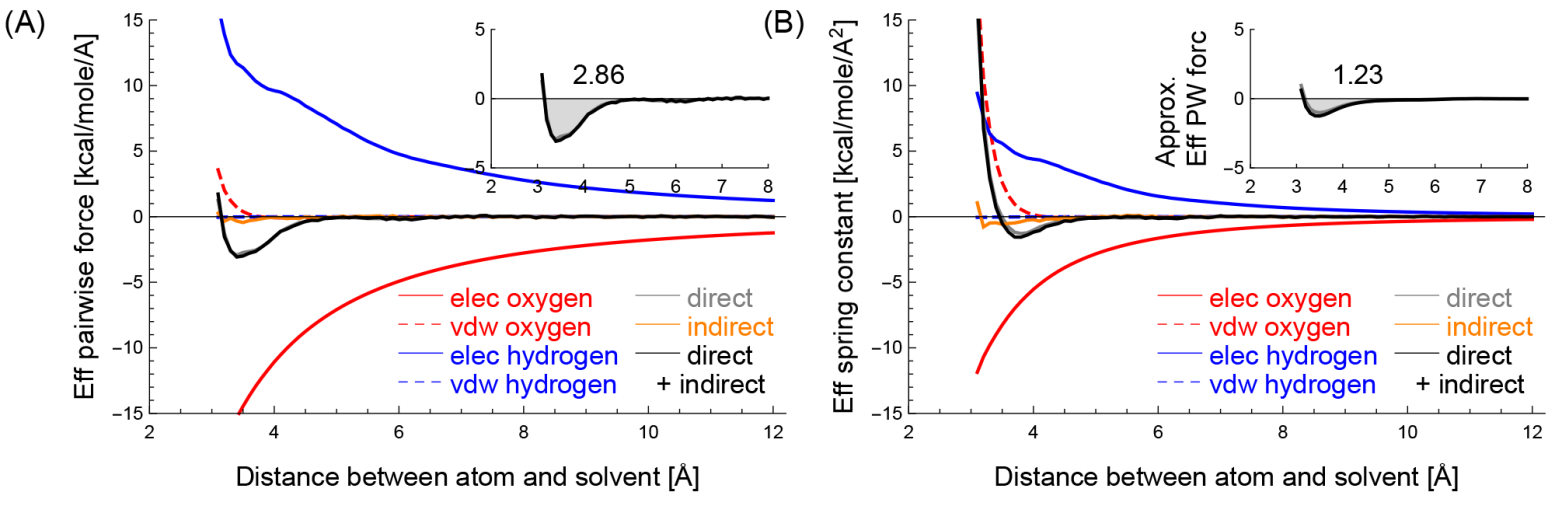
(A) The effective pairwise force and (B) the effective spring constant in the *y*-axis between the CZ carbon of Arg, which is hydrophilic, and a solvent particle decomposed into different components, when their distances are *x* Å. The inset in (A) shows black and gray curves, and the inset in (B) shows those approximated by using the black and gray curves in (B). The numbers in the insets show the gray areas.

**Figure 3:**
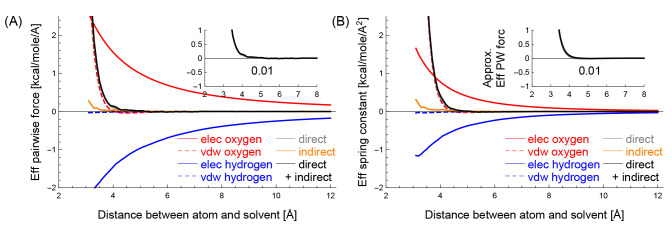
(A) The effective pairwise force and (B) the effective spring constant in the *y*-axis between the methine carbon, which is hydrophobic, and a solvent particle decomposed into different components, when their distances are *x* Å. Similarly to Figure 2, the inset in (A) shows black and gray curves, the inset in (B) shows their approximated curves, and the numbers in the insets show the gray areas.

Our results show that the effective pairwise forces and the effective spring constants can identify the effective hydrophilicity and hydrophobicity of atoms. Figures 2 and 3 show the statistics of different atom-solvent interaction types that help identify the hydrophilicity and hydrophobicity, respectively.

Figure 2(A) shows the mean effective pairwise force *f*_*ij*_ between the CZ carbon of Arg and a solvent particle, decomposed into the five components: *o*_*elec*_, *o*_*vdw*_, *h*_*elec*_, *h*_*vdw*_ and *s*_*indir*_. In the figure, the red curve represents the mean effective pairwise force determined by *o*_*elec*_ in the *y*-axis when the distance between the atom and the solvent particle is *x* Å. The mean effective pairwise force of *o*_*elec*_ at *x* Å is determined as follows: (i) select atom-solvent pairs whose distances are in [*x* – 0.05, *x* + 0.05) Å, (ii) calculate the effective pairwise force of *o*_*elec*_ component in eq 23 using eq 26 per pair, and then (iii) determine the average of the calculated values that lie between the fifth and 95th percentiles to exclude outliers. In a similar manner, the dashed red, blue, and dashed blue curves represent the mean effective pairwise forces of *o*_*vdw*_, *h*_*elec*_, and *h*_*vdw*_ components, respectively. The orange and gray curves represent those of the *s*_*indir*_ and *s*_*dir*_ components, which correspond to indirect and direct interactions, respectively, where *s*_*dir*_ = *o*_*elec*_ + *o*_*vdw*_ + *h*_*elec*_ + *h*_*vdw*_. The black curve represents those of the atom–solvent interaction, which is the sum of both direct and indirect interaction components: *s*_*all*_ = *s*_*dir*_ + *s*_*indir*_. The inset shows the black and gray curves and highlights the negative region of the black curve as a gray area. The number in the inset shows the value of the gray area.

In a similar manner, Figure 2(B) shows the mean effective spring constant *k*_*ij*_ between the CZ carbon of Arg and a solvent particle decomposed into the five components. The effective spring constant of each five component is determined by applying each five summand in eq 22 to eq 27. In contrast to the inset in Figure 2(A), the inset in Figure 2(B) shows the effective pairwise force *f̂*_*ij*_ approximated with the effective spring constant *k*_*ij*_ using the numerical integration, as follows:
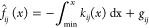
28where min is the smallest *x*-value in the plot, and *g*_*ij*_ is a constant value that makes the average of *f̂*_*ij*_(*x*) be zero for 8 ≥ *x* ≥ 12.

Similarly, Figure 3 panels (A) and (B) show the mean effective pairwise force *f*_*ij*_ and the mean effective spring constant *k*_*ij*_ between the methine carbon and a solvent particle, respectively, decomposed into the five components.

#### Analysis

Figures 2 and 3 show the effective hydrophilic and hydrophobic characteristics of the two atom types. Note that the force fields describe how an atom interacts with a water molecule (or a solvent particle) using nonbonded interactions. Among the nonbonded interactions, van der Waals interactions of the atom with the two water’s hydrogens *h*_*vdw*_ (dashed blue) are very weak compared to other interactions; the influences of *h*_*vdw*_ in the atom-water interaction can be almost neglected. The van der Waals interaction of the atom with the water’s oxygen *o*_*vdw*_ (dashed red) has a role in preventing steric clash. This is because *h*_*vdw*_ can be neglected, and the solvent’s location is equal to the oxygen’s location in this study. Therefore, the characteristics of the atom–water interactions, such as the hydrophilicity and hydrophobicity of the atoms, are mainly determined by electrostatic interactions *o*_*elec*_ + *h*_*elec*_.

When water molecules are coarse-grained to one-site solvent particles, it becomes unclear how to assign electrostatic interactions between a protein atom and a solvent particle. This is because each water molecule has zero net charge, so its electrostatic interaction vanishes. Figures 2 and 3 show that our method can assign the electrostatic interactions by separately considering the interaction with the water’s oxygen *o*_*elec*_ and those with the two water’s hydrogens *h*_*elec*_. Eventually, the effective hydrophilicity and hydrophobicity of the atoms can be determined, as explained below.

For example, on the one hand, Figure 2 shows that the CZ carbon of Arg has a *hydrophilic* characteristic. This is because there are pockets (gray-areas) between 3–5 Å in its two insets, and the pockets represent a potential energy well where a solvent particle can be trapped temporarily. This indicates that the atom attracts solvent particles statistically and can be classified as hydrophilic. On the other hand, Figure 3 shows that the methine carbon has the *hydrophobic* characteristic. This is because there are no such pockets (gray-areas) representing a potential energy well. Furthermore, its potential energy monotonically decreases as the distance between the atom and the solvent particle increases: the forces (black curves in the insets) are positive in all regions and monotonically decrease. This indicates that the atom constantly pulls or repulses solvent particles and can be classified as hydrophobic.

Note that the value in each inset shows how much the atom is hydrophilic. The value represents *the separation energy*: the maximum work required to separate the atom and the solvent particle (or to bring the solvent particle 12 Å from the atom in our calculation). We define the hydrophilic degree of an atom as the separation energy and calculate it by determining the averaged areas where the mean effective pairwise force *f*_*ij*_ and the approximated mean effective pairwise force *f̂*_*ij*_ are below zero. For example, the hydrophilic degree of the CZ carbon of Arg in Figure 2 is 2.045 and that of the methine carbon in Figure 3 is 0.01. Note that atoms with large (or small) hydrophilic degree values have effective hydrophilic (or hydrophobic) characteristics, which are net effects of water’s interacting preferences with the atoms and their covalently bonded neighbors.

Our experiment is related to the potential of mean force (PMF) as follows. Figures 2(A) and 3(A) are related to PMF in that they show ensemble-averaged forces between atoms and solvent particles. Additionally, the hydrophilic degree is related to PMF in that it measures the maximum work required to separate the atom and the solvent particle.

Figures 2 and 3 also show that the direct interaction *s*_*dir*_ is the key component in determining the effective hydrophilic or hydrophobic characteristic of an atom since the gray curves overlap with the black curves almost completely in both figures. This will be discussed further later.

### 3.3 Hydrophilic, Hydrophobic, and Amphipathic Characteristics of Amino Acids

Our results show that the hydrophilic, hydrophobic, and amphipathic characteristics of amino acids are accurately reproduced using the hydrophilic degree. We determine the hydrophilic degrees of all 71 atom types available in our data set. Table S1 in the Supporting Information lists the hydrophilic degrees of atom types determined using our method.

**Figure 4:**
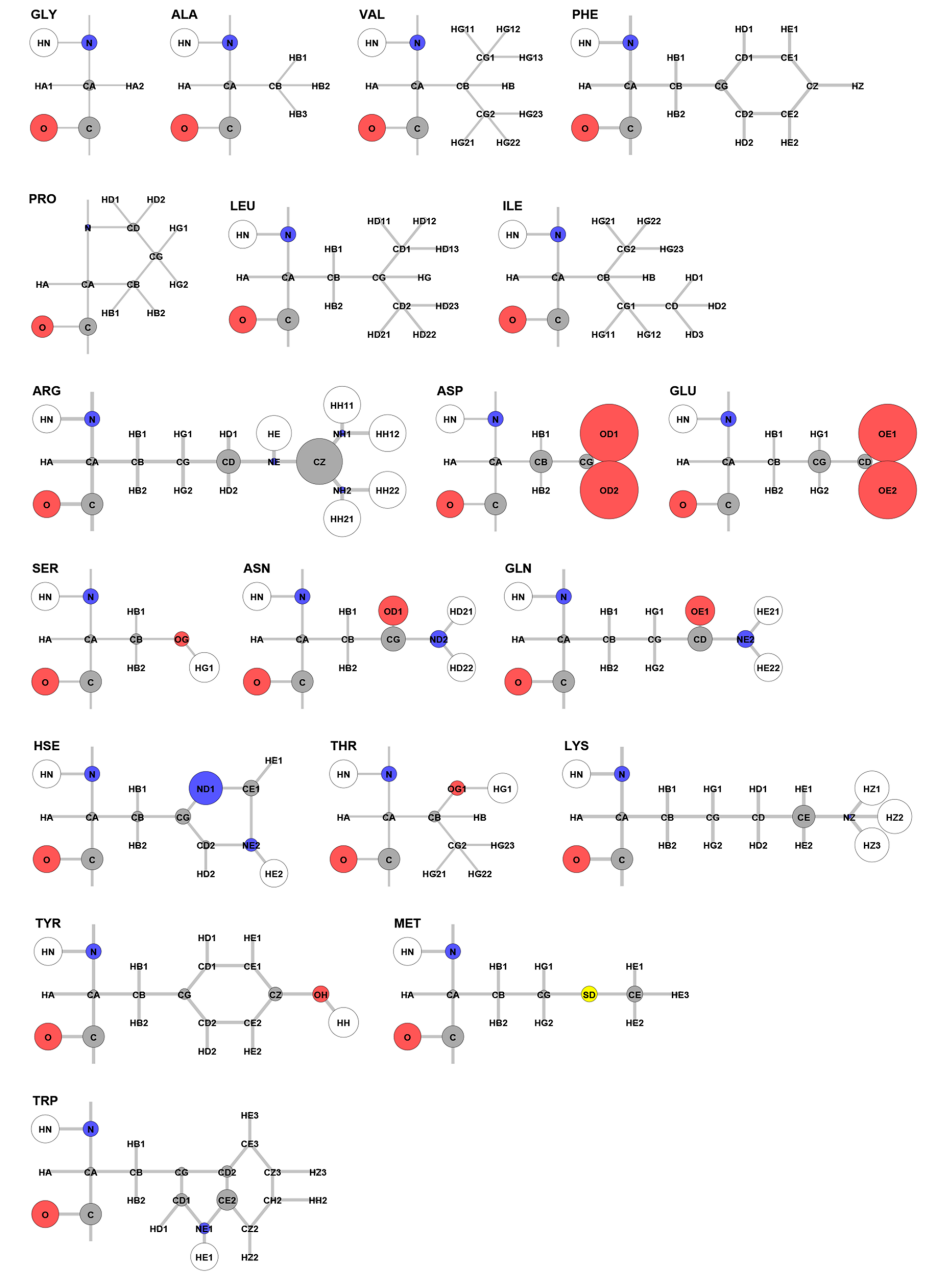
Hydrophilic degrees of atom types visualized on amino acid structures. The hydrophilic degrees are calculated with the 951 conformations collected from the 10 ns MD simulation of the sperm whale myoglobin (pdb-id: 1A6G). Cys is not included in the graphical representation because it is not present in 1A6G. The hydrophilic degrees may vary slightly if MD simulations of other proteins are used instead, but we anticipate that the overall hydrophilicity patterns in the amino acids remain the same.

Figure 4 visualizes the hydrophilic degrees of atom types on the amino acid structures. In the figure, the size of each circle is scaled to make its area proportional to the hydrophilic degree of its corresponding atom. Additionally, we make the areas of hydrogens 10 times smaller than those of heavy atoms. The figure does not include Cys because our data set collected from the sperm whale myoglobin does not have Cys. The results show that the characteristics of amino acids determined using our methods agree with those previously known and understood.^38,39^

When the hydrophilic degrees of atom types are calculated, Met CG is not properly calculated. This is because Met CG is buried under the protein surface in all of our MD conformations, as a result of which we do not collect enough statistics of the effective pairwise force and the effective spring constant in the short distance range. Considering its *h*_*elec*_ distribution pattern, its hydrophilic degree in Table S1 (and its radius in Figure 4) may need to be increased by around 2.5 times more than its current values (and 1.6 times larger than its radius). Except for Met CG and some Heme atoms, other atoms do not have the problem.

#### Backbone

The figure shows that backbone atoms are hydrophilic, especially for the chain of O=C–N–HN atoms in the peptide bonds, except for CA and HA. This is consistent for all residue types, even including hydrophobic amino acids. This is related to the dipole moments formed by peptide bonds.^39^ This implies that the O=C–N–HN atoms are favorable to interact with solvents, and thus, the solvents can assist and mediate the formation of backbone hydrogen bonds and secondary structures.

#### Hydrophobic Amino Acids

Side-chains of hydrophobic amino acids are known to have only van der Waals interactions. The figure shows that side-chain atoms of Ala, Val, Leu, and Ile are clearly hydrophobic: the hydrophilic degrees (areas of circles) of their side-chain atoms are close to zero. Even though the atoms have nonzero partial charges, their hydrophilic degrees are close to zero. Therefore, there is an aspect that the atoms interact with solvent particles mainly by using the van der Waals interactions. Side-chain atoms of Phe are close to hydrophobic except for CG that has a small hydrophilic degree value but is fully surrounded by hydrophobic atoms. This agrees with the observation that Phe is hydrophobic while its aromatic side chain can interact with waters weakly.^39^ Side-chain atoms of Pro are close to hydrophobic, but its CB, CD, and CG have small hydrophilic degree values; however, compared to other hydrophilic amino acids, Pro is almost hydrophobic.

#### Hydrophilic Amino Acids

In the figure, side-chain atoms of hydrophilic amino acids are obviously hydrophilic compared to those of hydrophobic amino acids. In our results, CZ of Arg, OD1/OD2 of Asp, OE1/OE2 of Glu, and ND1 of Hse are highly hydrophilic, which agrees with the characteristics of these amino acids. Interestingly, regarding Arg, the overall hydrophilic characteristic of its side-chain atoms is consistent with our understanding; however, its detail is somewhat different from our expectations and provides an interesting perspective in analyzing atom-solvent interactions. Arg has a positively charged guanidinium group. This explains why its positively charged atoms are hydrophilic: CZ (whose partial charge is 0.64 and hydrophilic degree is 2.05), CD (0.2 and 0.55), HE (0.44 and 11.93), and HH11–HH22 (0.46 and 13.19). However, even though its nitrogens have larger absolute partial charges than those of positively charged atoms, the nitrogens are nearly hydrophobic: NE (−0.7 and 0.04) and NH1/NH2 (−0.8 and 0.04). This is somewhat counterintuitive but can be explained as follows: because the guanidinium group is positively charged, oxygens of waters near the guanidinium group tend to be located closer to the group than hydrogens do. As a result, positively charged atoms become strongly hydrophilic, while negatively charged atoms tend to be hydrophobic.

#### Amphipathic Amino Acids

In the figure, side-chains of Thr, Lys, Tyr, Met, and Trp have both hydrophobic and hydrophilic parts, which are typical characteristics of amphipathic amino acids. Both Thr and Tyr have a hydrophilic −O–H group, while the rest of their side-chain atoms are hydrophobic. Tyr has an aromatic ring that can interact with water molecules weakly. In a similar manner, Trp has a hydrophilic −N–H group, and its nearby atoms, including CD1/CD2 and CE2, are also hydrophilic. Regarding Lys, the overall hydrophilic/hydrophobic characteristic of its side-chain atoms is consistent with our understanding; however, its detail is somewhat different from our expectations, similar to Arg. It is expected that the hydrophilic characteristic of Lys’s end is contributed mainly by NZ (whose partial charge is −0.3) and HZ1/HZ2/HZ3 (0.33) rather than CE (0.2) since the magnitude of the partial charge of CE is smaller than the others. However, our results show that the hydrophilic characteristic is mainly contributed by CE (whose hydrophilic degree is 0.49) and HZ1/HZ2/HZ3 (11.37), while NZ (0.01) is close to hydrophobic.

### 3.4 Roles of Water’s Hydrogens in Interacting with Two Hydrophilic and Hydrophobic Atoms

Figures 2 and 3 show that the effective hydrophilicity and hydrophobicity of atoms can be distinguished using the mean effective pairwise force and the mean effective spring constant. Our investigation shows that the water’s hydrogens have two roles in atom–solvent interactions. First, the statistical preferences of the hydrogens’ relative locations from their water center determine the effective hydrophilicity and hydrophobicity. Second, the collective hydrogen dynamics assist solvent particles in escaping from the mean potential energy wells of the hydrophilic atoms. We explain this in detail in the following.

**Figure 5:**
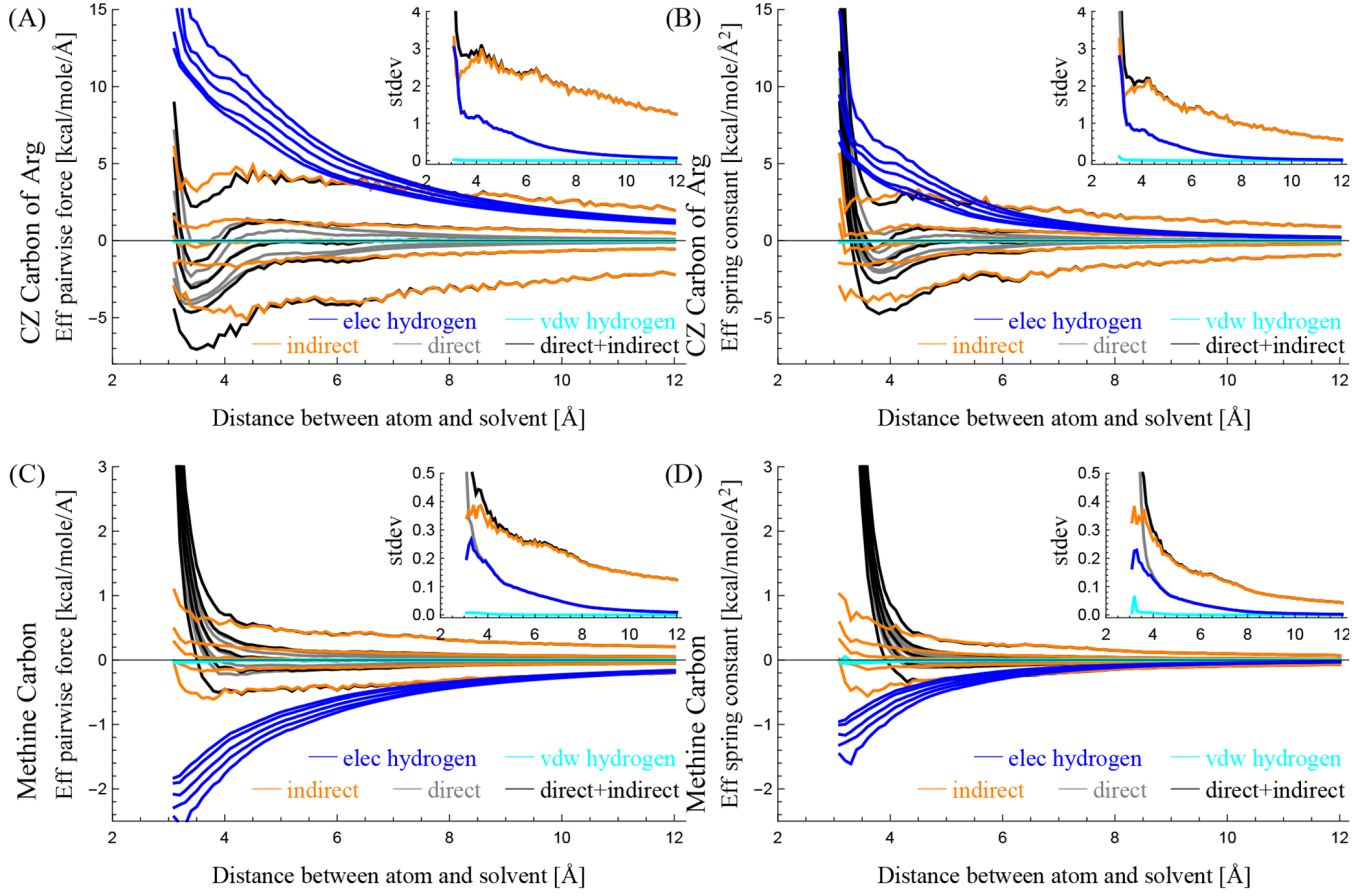
Detailed statistics of the CZ carbon of Arg and the methine carbon that are not present in Figures 2 and 3, respectively. Panels (A) and (C) show the detailed statistics of the effective pairwise forces, and (B) and (D) show the detailed statistics of the effective spring constants. The five curves with the same color show the 10th, 25th, 75th, and 90th percentiles and the mean value of their corresponding interactions. The inset shows the standard deviations of the interactions.

Recall that the mean effective pairwise force (and the mean effective spring constant) between an atom and a solvent particle is decomposed into four direct interactions and one indirect interaction: *o*_*elec*_ + *o*_*vdw*_ + *h*_*elec*_ + *h*_*vdw*_ + *s*_*indir*_. Among the five interactions, the following three interactions cannot be determined from the distance between the atom and the solvent particle: *h*_*elec*_, *h*_*vdw*_, and *s*_*indir*_. The indirect interaction *s*_*indir*_ is determined by mediation of the collective hydrogen dynamics. Two direct interactions with hydrogens *h*_*elec*_ and *h*_*vdw*_ are determined from two hydrogens’ relative locations from the water center (or simply hydrogens’ poses), such as atom-hydrogen distances and hydrogen-atom-oxygen angles for the projection in eqs 26 and 27. Note that the atom-solvent interaction types, such as hydrophilic and hydrophobic, are characterized by the above three interactions since *o*_*elec*_ and *o*_*vdw*_ are determined from the atom–solvent distance. Figure 5 shows the roles of *h*_*elec*_, *h*_*vdw*_, and *s*_*indir*_, which will be explained in detail at the end of this section.

Figure 5 shows the additional statistics of the effective pairwise forces and those of the effective spring constants that are not presented in Figures 2 and 3. In Figure 5(A), the five blue (and cyan and orange) curves show the 10th, 25th, 75th, and 90th percentiles and the mean value of the effective pairwise forces of *h*_*elec*_ (and *h*_*vdw*_ and *s*_*indir*_). The middle blue (cyan and orange) curve is the same as the blue (dashed blue and orange) curve in Figure 2(A). In a similar manner, the five gray curves show those of the direct interaction *s*_*dir*_ that is the sum of all direct interactions, and their middle curve is the same as the gray curve in Figure 2(A): *s*_*dir*_ = *o*_*elec*_ + *o*_*vdw*_ + *h*_*elec*_ + *h*_*vdw*_. The five black curves show those of the effective pairwise force of the atom-solvent interaction *s*_*all*_ that is the sum of both the direct and indirect interactions, and the middle black curve is the same as the black curve in Figure 2(A): *s*_*all*_ = *s*_*dir*_ + *s*_*indir*_. The inset shows the standard deviations of the five interactions, determined from values between the fifth and 95th percentiles. In a similar manner, Figure 5(B) shows the additional statistics of the effective spring constants not presented in Figure 2(B). Similarly, Figure 5 panels (C) and (D) show the additional statistics not presented in Figure 3 panels (A) and (B), respectively.

#### The Statistical Preference of Hydrogens’ Poses

Figure 5 shows that one of the roles of hydrogens is to determine the effective hydrophilic or hydrophobic characteristic of a protein atom. In all plots, the middle blue curves (the mean value of *h*_*elec*_) deviate from the *x*-axes while the middle cyan and the middle orange curves (those of *h*_*vdw*_ and *s*_*indir*_) are superimposed onto the *x*-axes. Recall that the direct interaction *s*_*dir*_ is composed of *o*_*elec*_, *o*_*vdw*_, *h*_*elec*_, and *h*_*vdw*_, where *o*_*elec*_ and *o*_*vdw*_ are determined from the atom–solvent distance and *h*_*elec*_ and *h*_*vdw*_ are determined by the hydrogens’ poses (the hydrogens’ relative locations from the water center). This indicates that the statistical preference of hydrogens’ poses determines first the mean value of *h*_*elec*_ (the middle blue curves), then the mean effective pairwise force and the mean effective spring constant (the middle black curves), and consequently the effective hydrophilic or hydrophobic characteristic of the atom. This implies that the statistical preference of hydrogens’ poses is the key component in determining a protein atom’s effective hydrophilicity and hydrophobicity.

#### The Collective Hydrogen Dynamics

Figure 5 shows that the other role of hydrogens is to assist solvent particles in escaping from the mean potential energy well of hydrophilic atoms. In all plots, the gap/deviation between the top and bottom orange curves (the 10th and 90th percentiles of *s*_*indir*_) is wider than that of blue curves (those of *h*_*elec*_), and the top and bottom orange curves are well superimposed onto those of black curves (those of *s*_*all*_) especially when *x* > 5 Å. This is reflected in its inset: the *y*-value of the orange curve (the standard deviation of *s*_*indir*_) is greater than that of the blue curve (that of *h*_*elec*_), and the orange curve (*s*_*indir*_) is almost superimposed onto the black curve (*s*_*all*_). This indicates that *s*_*indir*_ is the major source of deviating the effective pairwise force and the effective spring constant from their mean values, even though *h*_*elec*_ partially contributes to the deviation. Note that the top black curves (the 90th percentile of *s*_*all*_) in Figures 5 panels (A) and (B) are located even fully above the *x*-axis, indicating that the CZ carbon of Arg can become hydrophobic temporarily even though it is considered as hydrophilic based on its mean effective pairwise force and spring constant. Recall that *s*_*indir*_ is determined by the mediation of the collective hydrogen dynamics. This implies that the collective hydrogen dynamics assists solvent particles in escaping from the mean potential energy wells of hydrophilic atoms by making the wells shallower or by making them hydrophobic temporarily.

Note that the middle orange curves (the mean value of *s*_*indir*_) are superimposed onto the *x*-axes in all plots of Figure 5. This additionally implies that the collective hydrogen dynamics do not significantly influence the mean potential energy surfaces of the atom–solvent interactions since the deviations in *s*_*indir*_ cancel out themselves and vanish.

### 3.5 Roles of Water’s Hydrogens in Interacting with All Other Atom Types

**Figure 6:**
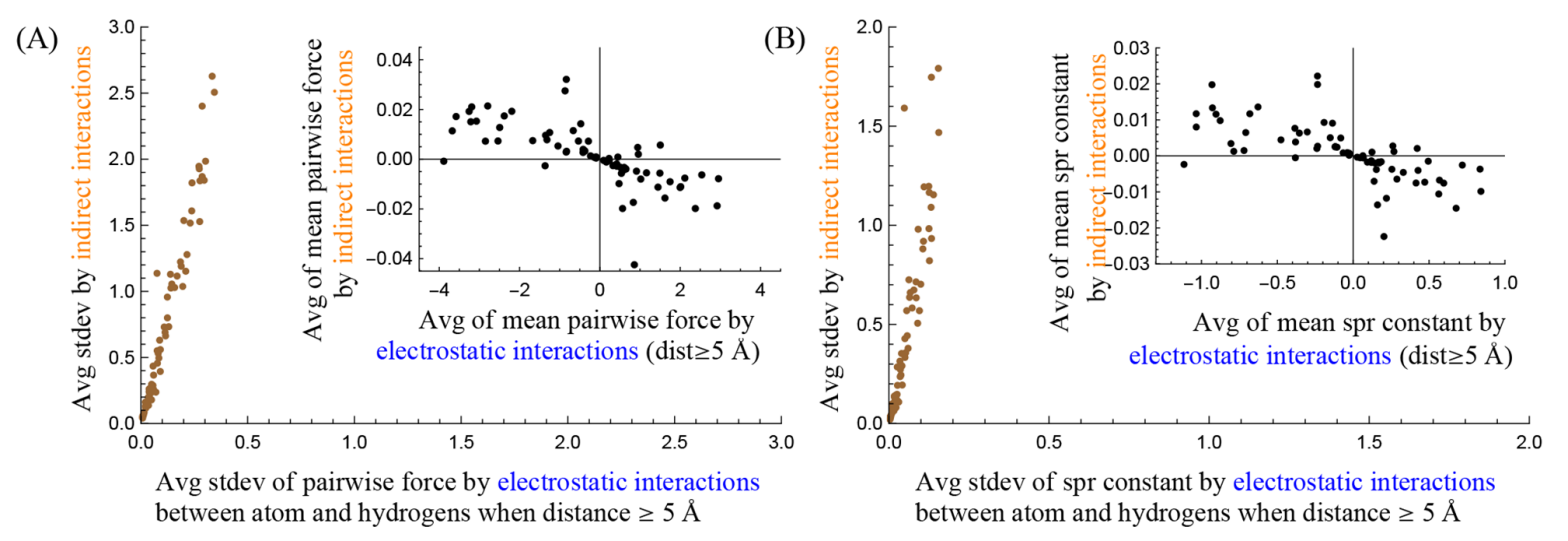
Comparison of the deviation statistics of *h*_*elec*_ in the *x*-axis and *s*_*indir*_ in the *y*-axis of all atom types. In (A), each black (and brown) point represents the average of an atom type’s mean effective pairwise force (and the standard deviation of the effective pairwise force) of *h*_*elec*_ in the *x*-axis and *s*_*indir*_ in the *y*-axis. Similarly, (B) shows those of the effective spring constant.

Figure 5 gives insights into the roles of hydrogens from two sample hydrophilic and hydrophobic atoms. Figure 6 generalizes the insights to all other atom types.

Figure 6 shows deviation statistics of *h*_*elec*_ and *s*_*indir*_ of all atom types. Figure 6(A) compares the statistics of the effective pairwise force of *h*_*elec*_ in the *x*-axis and those of *s*_*indir*_ in the *y*-axis. In the figure, each black point represents an atom type whose *x* and *y* values are the average of the mean pairwise force of *h*_*elec*_ and that of *s*_*indir*_, respectively, given that the distance between the atom and a solvent particle is between 5 and 12 Å. Similarly, each brown point represents an atom type whose *x* and *y* values are the average of the standard deviation of the pairwise force of *h*_*elec*_ and that of *s*_*indir*_, respectively. For example, Figure 5 panel (A) or (C) (and its right-top inset) becomes a black (and brown) point whose *x* and *y* values are calculated by averaging the *y* value of the middle blue and middle orange curves (and the blue and orange curves) averaged over 5 and 12 Å, respectively. In a similar manner, Figure 6(B) compares the statistics of the effective spring constants of *h*_*elec*_ and those of *s*_*indir*_.

The distributions of black points in Figure 6 panels (A) and (B) show that the *x* value is around 120 and 50 times bigger than the *y* value in average, respectively. This indicates that the deviations of the mean effective pairwise force and spring constant of *h*_*elec*_ from zero value are around 85 times bigger than those of *s*_*indir*_. This generalizes our observation that the statistical preferences of hydrogen poses are the key components in determining the effective hydrophilicity and hydrophobicity of the atoms. Additionally, *y* values of all black points are very close to zero. This generalizes our observation that the collective hydrogen dynamics do not significantly influence the mean atom-solvent interactions since the deviations in *s*_*indir*_ cancel out themselves and vanish.

The distributions of brown points in Figure 6 panels (A) and (B) show that the *y* value is around 6.3 and 7.8 times bigger than the *x* value in average, respectively. This indicates that the standard deviations of the effective pairwise force and spring constant of *s*_*indir*_ are around 7 times bigger than those of *h*_*elec*_. This generalizes our observation that the collective hydrogen dynamics assists solvent particles in escaping from the mean potential energy wells of hydrophilic atoms.

### 3.6 Influence of the Viscous Behavior of Solvents on the Dynamics of Protein Atoms

Dynamics (especially fast and high-frequency dynamics) of proteins are coupled with the dynamics of solvent,^40^ while their secondary structures and their slow- and low-frequency dynamics are less sensitive to solvent dynamics.^41^ To include the influence of solvents in studying protein dynamics,^42−45^ often the Langevin mode analysis^46^ that models the dynamics of proteins immersed in a viscous solvent has been used; a uniform viscosity value has been used additionally to determine the friction matrices of the proteins.^44,47^ Our study shows that the bulk solvents can be modeled using a uniform viscosity value when describing their influence on protein dynamics. However, modeling the hydration shell using the same uniform viscosity value may have a limitation and cause inaccuracy in describing the dynamics of proteins in solvents.

Here, we evaluate the influence of solvents as a viscous medium on protein dynamics by inspecting the motion correlation *C*_*a*,*b*_ (or cooperativity) between a protein atom *a* and a solvent particle *b*. The motion correlation is calculated as follows:^21^
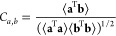
29where **a** and **b** are the displacement vectors for *a* and *b* in a given mode, respectively, and ⟨**x**^T^**y**⟩ is the average value of dot product of two vectors **x** and **y** weighted by their eigenvalues. We consider that the modes with negative eigenvalues also contribute to the motion of the system, even though they are unstable modes. To involve the unstable modes, we calculate ⟨**x**^T^**y**⟩ as follows:
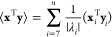
30where *n* is the total number of modes, **x**_*i*_ and **y**_*i*_ are the displacement vector **x** and **y** in the *i*th mode, respectively, λ_*i*_ is the eigenvalue of the *i*th mode, and λ_*j*_ = 0 for all 1 ≤ *j* ≤ 6.

**Figure 7:**
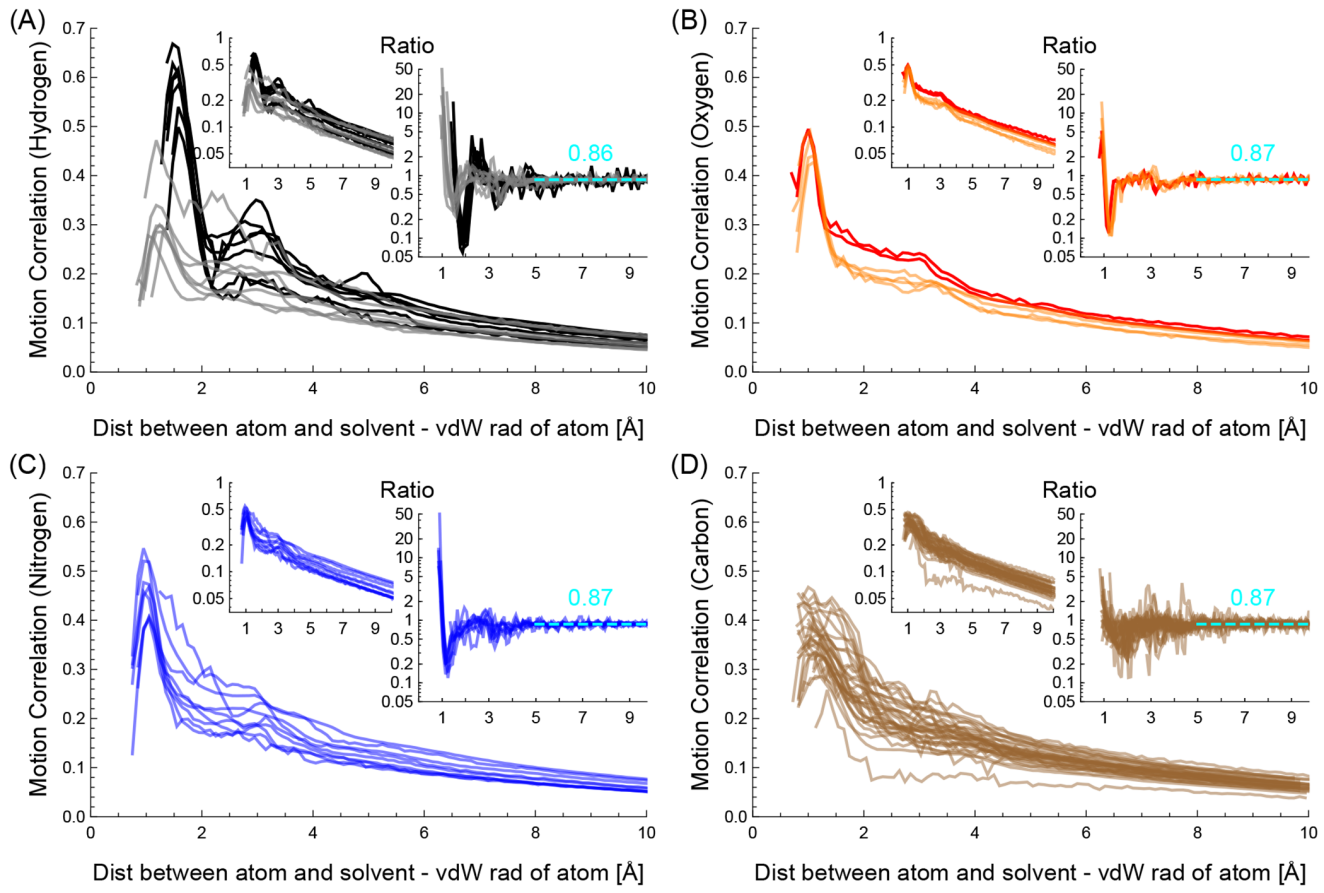
Motion correlation between solvent particles and (A) hydrogen, (B) oxygen, (C) nitrogen, or (D) carbon types. The black curves in (A) and the red curves in (B) show those of hydrogen and oxygen types whose hydrophilic degree values are greater than 3, respectively.

Figure 7 shows the motion correlation between the different atom types and solvent particles. In Figure 7(A), each gray curve shows the mean motion correlation between its corresponding protein’s hydrogen and a solvent particle when their distance is (*x* + *r*) Å, where *r* is the van der Waals radius of the protein’s hydrogen. The mean motion correlation at *x* Å is determined by calculating the average of the motion correlations that lie between the fifth and 95th percentiles to remove outliers. The middle-top inset shows the same plot on a logarithmic scale. The right-top inset shows the ratio *r* of the motion correlation, which is determined using the slope in logarithmic scale, as follows:
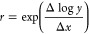
31where *x* and *y* are the distance and the mean motion correlation, respectively. The ratio *r* approximates that of *y* = *ar*^*x*^ + *b*. The number in the right-top inset shows the average ratio when *x* ∈ [5, 10] Å. In a similar manner, the orange, blue, and brown curves in Figure 7 panels (B), and (C), and (D) show the mean motion correlations of oxygen, nitrogen, and carbon types, respectively. The black and red curves in Figure 7 panels (A) and (B) show those of the seven hydrogen and two oxygen types whose hydrophilic degree values are greater than 3, respectively.

The figures show three interesting viscosity properties of solvents. Note that the hydration shell is the group of solvent particles next to the surface of proteins within about 3.5 Å,^48^ and the bulk solvents are the group of solvent particles located outside of the hydration shell. First, the interactions between the protein atoms and bulk solvents can be modeled using a uniform viscosity value of the solvent. The middle-top insets of Figure 7 show that the motion correlations decrease near-exponentially as the distances between protein atoms and solvent particles increase: the motion correlations in logarithmic scales decrease linearly. The right-top insets of the figures show that all their ratios are nearly 0.87, especially when *x* > 5 Å. The exponential decreases in motion correlations between protein atoms and solvent particles are related to the solvent viscosity. Viscosity is often referred to as fluid friction and is defined by using the ratio of speed change by the distance change. In our system, we consider that the viscosity of solvent is related to the ratio of motion correlation change by the distance change, which is equal to the slope on the logarithmic scale. This observation implies that the bulk solvents can be modeled using a uniform viscosity value independent of protein atom types.

Second, the viscosities of the hydration shell and bulk solvents may need to be treated differently. Figure 7 shows peaks and drastic drops of the motion correlation near the protein surface, such as 1–2 Å, compared with those in bulk solvents. The drastic drop is caused while bridging the gap between the motion correlation on the protein surface and that on the bulk solvents. The insets show the increase of slopes of curves in the same region. Reproducing the drastic drop and the slope increase can be achieved by introducing the effective viscosity of the hydration shell that is lower than the viscosity of bulk solvents. This implies that different viscosity values may need to be used for the hydration shell and the bulk solvents.

Third, strongly hydrophilic atom types have different characteristics in interacting with solvent particles compared to other atom types. Let us consider that atoms are strongly hydrophilic if their hydrophilic degree values are greater than 3. In the figure, the black and red curves represent the protein’s hydrogens and oxygens that are strongly hydrophilic, respectively. Note that the black and red curves show different motion correlation patterns in the hydration shell, where *x* < 3.5 Å, compared to the gray and orange curves. This implies that solvents interact differently with strongly hydrophilic atoms than with other atoms.

### 3.7 Interactions between Solvent Particles

Even though our experiments are designed to observe the statistics of atom–solvent interactions, we additionally collect the statistics of solvent–solvent interactions. Our results show that the statistics are in agreement with others’ observations.

**Figure 8:**
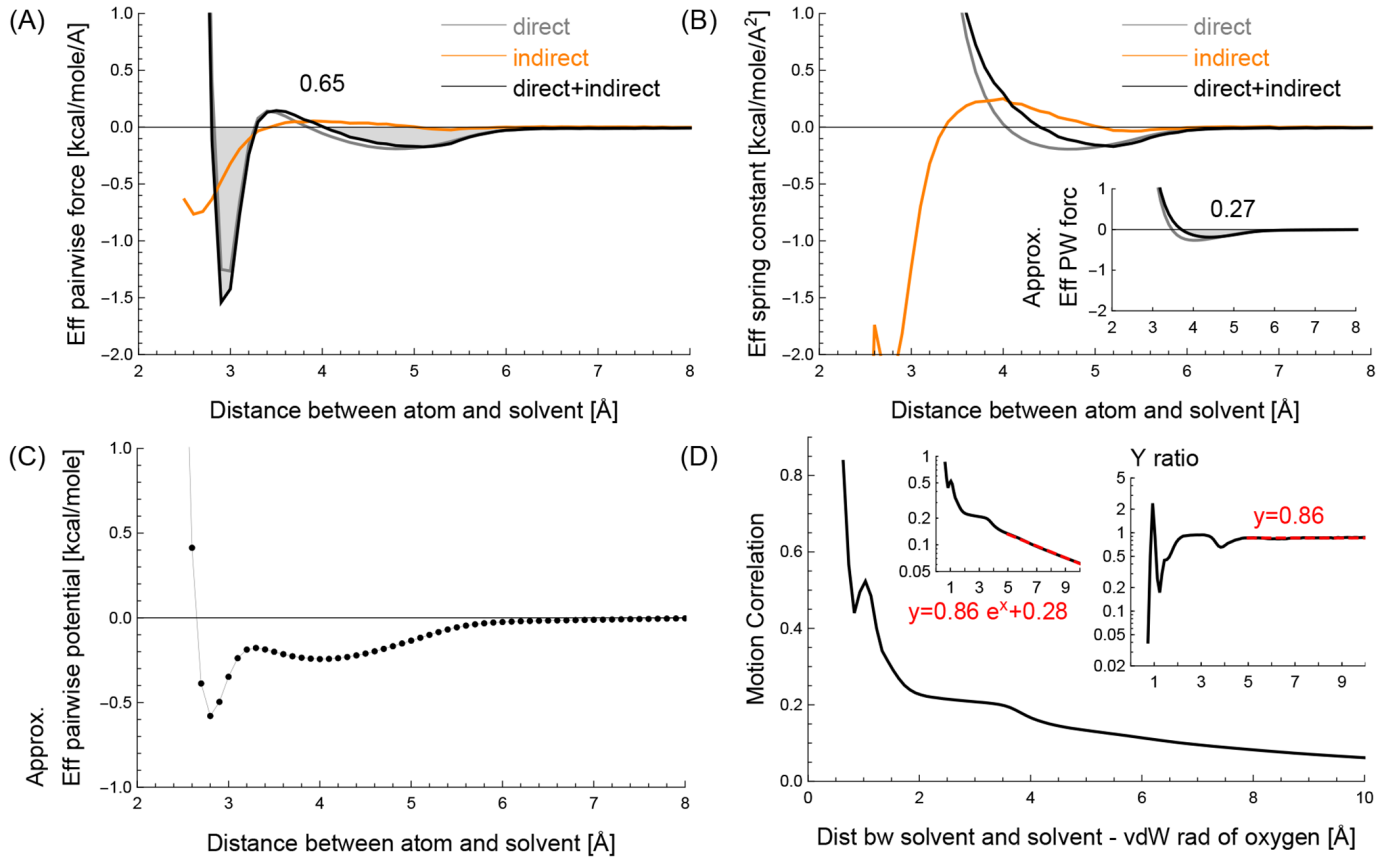
(A) The effective pairwise force and (B) the effective spring constant in the *y*-axis between two solvent particles when their distance is *x* Å. The number in each plot shows the gray area that highlights the negative region of the black curve. (C) The numerical integration of the effective pairwise force in (A). (D) The motion correlation between solvent particles.

Figure 8 panels (A) and (B) show the mean effective pairwise force and the mean effective spring constant between two solvent particles, respectively. In the figures, the gray and orange curves represent the direct interaction *s*_*dir*_ and the indirect interaction *s*_*indir*_, respectively, and the black curve represents the sum *s*_*all*_ of the direct and indirect interactions. The numbers on the figures show the gray areas, where the mean effective pairwise force *f*_*ij*_ and the approximated mean effective pairwise force *f̂*_*ij*_ are below zero. Figure 8(C) shows the potential energy graph built using the numerical integration in eq 28 and the black curve in Figure 8(A). In Figure 8(C), there is a local minimum between 2.7 and 2.8 Å. This has an agreement with the peaks observed from the radial distribution function of the all-atom TIP3P.^37,49,50^

Figure 8(D) shows the motion correlation between the two solvent particles. The dashed red line and the number in the right-top inset show the average ratio when *x* ∈ [5, 10] Å. The ratio of 0.86 in the right-top inset closely matches the ratios in Figure 7. This implies that a uniform viscosity value can be used for the bulk solvents independent of its interacting partners, whether they are protein atoms or solvent particles, when the interacting distances are greater than 5 Å.

## 4 Conclusions

In this work, we propose a new method for coarse-graining water molecules and extracting pairwise forces and spring constants of interactions between protein atoms and coarse-grained water molecules. Our coarse-graining method is developed based on the coarse-graining approaches used in the normal mode analysis and the elastic network models^21−23^ and by utilizing the method of coarse-graining the potential energy surface of non-minimized systems.^32^ Our method considers each coarse-grained unit (a water molecule in this work) as a whole unit (a solvent particle) and coarse-grains the relative changes of its elements (namely, the relative locations of the water’s oxygen and hydrogens from the center of solvent particle). We call this method the coarse-graining-delta or δ-CG in short. This enables us to describe electrostatic and van der Waals interactions of both waters’ oxygens and hydrogens using only the solvent particles’ locations. We then determine the effective pairwise forces and the effective spring constants between protein atoms and solvent particles.

After proposing the method, we demonstrate the method for extracting and inspecting components of the effective pairwise forces and the effective spring constants. Our results provide several insights into how the physicochemical characteristics of protein atoms are decided by interactions with solvent particles. First, the hydrophilic and hydrophobic characteristics of protein atoms and amino acids can be identified by using our hydrophilic degree that measures the maximum work required to separate a protein atom and a solvent particle. The hydrophilic degree reveals the effective hydrophilicity and hydrophobicity of protein atoms even though a water molecule has zero net charge. Additionally, the hydrophilic, hydrophobic, and amphipathic characteristics of amino acids can be accurately reproduced using the hydrophilic degree.

Second, water’s hydrogens have roles in deciding the effective hydrophilicity and hydrophobicity of protein atoms and in assisting waters in escaping from the hydrophilic atoms. The effective pairwise forces and the effective spring constants can be decomposed into direct and indirect interactions. The direct and indirect interactions are determined from the statistical preference of water’s (or its hydrogens’) pose and the collective dynamics of waters’ hydrogens, respectively. Our results show that (i) the statistical preference of the hydrogens’ poses is the key to determining the effective hydrophilicity and hydrophobicity of atoms and (ii) the collective hydrogen dynamics are the major contributor in assisting solvent particles to escape from the potential energy wells of hydrophilic atoms.

Finally, the bulk solvents can be modeled as a viscous medium having a uniform viscosity value independent of its interacting partners. The viscosity of the hydration shell may be different from that of the bulk solvents. Our result suggests that the effective viscosity value of the hydration shell can be smaller than that of bulk solvents.

Our results are collected only from wild-type sperm whale myoglobin; therefore, the results may be biased. However, considering that about 2,500 atoms of myoglobin are grouped into 71 subtypes, we expect that even if additional experiments are performed using more proteins, the results will be similar to our results. Adding salt ions into the system can increase the stability of some proteins.^51^ However, our system does not have salt ions since myoglobin is able to maintain its structural stability without salt ions. Additionally, this is because the system without salt ions can be used as a benchmark to evaluate how much the interaction between protein atoms and water molecules changes with/without salt ions.

### Implication

Our study and results have three implications. First, our study shows that coarse-graining a system breaks the mathematical antisymmetry of pairwise forces between its units even though the pairwise forces of the original system are antisymmetric. It looks counterintuitive at first glance, since this implies that the two interacting units exert different forces on each other. However, this can be explained if we consider that the coarse-graining process loses information, the loss is compensated in other forms (such as indirect interactions), and the compensation does not guarantee mathematical antisymmetry. This implies that when describing the dynamics of systems using coarse-grained force fields, the pairwise forces between any two coarse-grained units may need to be determined using different formulas to improve the accuracy of the dynamics. This may be especially crucial for running molecular dynamics simulations using the course-grained force fields that extensively coarse-grain large building units, such as the MARTINI force field^1−5^ and the UNRES force field.^33,34^

Second, our results show the potential of developing a coarse-grained water/solvent model in a new way. The effective pairwise force and the effective spring constant between a protein atom and a solvent particle are decomposed into direct and indirect interactions. The direct interaction is decomposed again into atom-oxygen and atom-hydrogen interactions. The atom-oxygen interaction is determined from the atom-solvent distance. The atom-hydrogen interaction makes the atom hydrophilic or hydrophobic when combined with the atom-oxygen interaction. The indirect interaction statistically deepens or shallows the potential energy wells of the hydrophilic atoms. This implies that we can develop a single-point solvent model that accurately predicts protein–solvent interactions by approximating the mean atom–hydrogen interaction and the statistics of the indirect interaction. The model can have obvious strengths compared to existing coarse-grained water models, including (i) accurately describing the interactions between proteins and solvents at the atomic level, compared to one-site models,^8−11^ (ii) saving computational resources, compared to two-site models,^8,12−14^ and (iii) handling each solvent particle independently, compared to cluster models.^4,15−20^ The strength (iii) is especially useful in describing solvent-mediated protein–protein interactions since the model can properly describe individual water molecules that are trapped inside the biomolecules or located in a thin layer at the interface of two biomolecules.

Last, we propose a new systematic approach of coarse-graining a system and extracting the pairwise forces and the spring constants. This approach is based on the normal mode analysis and uses the potential energy surface of the system. Therefore, this approach makes it possible to inspect and study the roles of coarse-grained unit’s interaction components by observing their statistics in the potential energy surface, instead of observing the molecular-dynamics trajectories as employed in other approaches. In our knowledge, this type of study has not been done widely. This approach may provide new insights into coarse-grained systems and/or the role of building units in biomolecules. Additionally, the approach can provide a new way of studying the distinctions of different water models, such as TIP3P, TIP4P, and TIP5P water models.^37,52,53^

In this work, we demonstrated how much coarse-graining water molecules into single-point solvent particles is useful in determining the effective pairwise forces and the effective spring constants between protein atoms and waters and in identifying the hydrophilicity/hydrophobicity of the atoms. The proposed coarse-graining method can be used in developing and refining coarse-grained force fields, such as the MARTINI force field^1−5^ and the UNRES force field,^33,34^ in deciding the groups of atoms to be coarse-grained as well as determining the coarse-grained potential energy functions and their coefficients. This can be done by inspecting the interaction components between coarse-grained units in the coarse-grained potential energy surface, as we did to the electrostatic and van der Waals interactions between atom-solvent interactions in this work. More specifically, the energy functions and their coefficients can be designed to predict the effective pairwise forces for the molecular dynamics simulations or the effective spring constants for the normal mode analyses. The same approach can be used to refine coefficients of coarse-grained force fields, especially coefficients of amino acids in different secondary structure settings, which may improve the accuracy of molecular dynamics simulation results.

## Supporting Information Available

Hydrophilic degrees of atom types (PDF)
