# Supplementary material for: Coarse-Graining Waters: Unveiling The Effective Hydrophilicity/Hydrophobicity of Individual Protein Atoms and The Roles of Waters’ Hydrogens
Source: J Chem Theory Comput. 2023 Oct 2;19(20):7307–23. doi: 10.1021/acs.jctc.3c00700 (PMC10601925; doi:10.1021/acs.jctc.3c00700)
Supplement: Supplementary file 1 — ct3c00700_si_001.pdf [file ct3c00700_si_001.pdf]

Supporting information:
Coarse-Graining Waters: Unveiling The Effective Hydrophilicity/Hydrophobicity of Individual Protein Atoms and The Roles of Waters' Hydrogens

Hyuntae Na[a,*], Guang Song[b]

[a] Department of Computer Science, Penn State Harrisburg, Middletown, Pennsylvania, USA

[b] Department of Mathematics and Computer Science, Westmont College, Santa Barbara, California, USA

- Table S1: The hydrophilic degrees of atom types

Table S1: The hydrophilic degrees of atom types, grouped by backbone atoms, carbons, hydrogens, nitrogens, oxygen, and sulfur. This table includes 63 atom types from the total of 71 atom types collected from our dataset, excluding Heme atoms.

Description	Hydrophilicity			Description	Hydrophilicity		
	spr[a]	frc[b]	avg[c]		spr[a]	frc[b]	avg[c]
N	0.13	0.32	0.23	MET CG[d]	0.02	0.10	0.06
CA	0.07	0.06	0.07	Aromatic Carbon	0.00	0.01	0.01
C	0.46	0.45	0.46	Methine Carbon	0.01	0.01	0.01
O	0.01	1.43	0.72	Methyl Carbon	0.00	0.00	0.00
HN	5.89	9.38	7.63	Methylene Carbon	0.01	0.00	0.01
HA	0.11	0.12	0.11	ARG HH	10.88	15.5	13.19
GLY CA	0.10	0.11	0.10	ARG HE	9.16	14.70	11.93
PRO CA	0.07	0.08	0.07	LYS HZ1/HZ2/HZ3	9.61	13.12	11.37
PRO N	0.04	0.06	0.05	Hydroxyl Hydrogen	4.92	12.37	8.65
ARG CZ	1.23	2.86	2.05	HSE Ring HE2	5.62	9.27	7.45
N-Terminal CA	0.44	1.09	0.77	TRP Indole HE1	5.13	9.50	7.31
ASN CG/GLN CD	0.53	0.63	0.58	N-Terminal HCA	0.77	1.18	0.98
ARG CD	0.39	0.71	0.55	HSE Ring HE1	0.19	0.46	0.32
LYS CE/ORN CD	0.32	0.67	0.49	LYS HE	0.24	0.32	0.28
ASP CB/GLU CG	0.36	0.61	0.48	Aromatic Hydrogen	0.25	0.31	0.28
TRP CE2	0.37	0.46	0.42	Nonpolar Hydrogen	0.21	0.26	0.24
MET CE	0.17	0.33	0.25	HSE HD2	0.18	0.29	0.23
HSE Ring CE1	0.25	0.23	0.24	HSE Ring ND1	0.00	2.16	1.08
HSE Ring CG	0.23	0.23	0.23	ASN ND2/GLN NE2	0.14	0.43	0.29
ASP CG/GLU CD	0.16	0.25	0.20	HSE Ring NE2	0.13	0.20	0.16
TYR CZ	0.19	0.18	0.18	TRP Pyrrole NE1	0.01	0.22	0.12
TRP CD1	0.14	0.17	0.16	ARG NE	0.00	0.09	0.04
TRP CD2	0.11	0.15	0.13	ARG NH	0.00	0.07	0.04
THR CB	0.10	0.13	0.12	LYS NZ, Ammonium Nitrogen	0.00	0.03	0.01
HISE CB	0.08	0.14	0.11	C-Terminal O	1.39	5.28	3.34
PHE/TYR CG	0.11	0.11	0.11	ASP OD/GLU OE	0.98	5.56	3.27
SER CB	0.11	0.11	0.11	ASN OD1/GLN OE1	0.01	1.67	0.84
PRO CD	0.10	0.10	0.10	Phenol Oxygen	0.01	0.51	0.26
C-Terminal C	0.04	0.13	0.08	Hydroxyl Oxygen	0.01	0.41	0.21
HSE Ring CD2	0.07	0.07	0.07	Sulfide Sulfur	0.21	0.27	0.24
TRP CG	0.07	0.07	0.07	TIP3P Oxygen	0.27	0.65	0.46
PRO CB and CG	0.07	0.06	0.07				

[a]The area where the approximated mean effective pairwise force is below zero, which is determined by integrating the effective spring constant; [b]The area where the mean effective pairwise force is below zero; [c]Hydrophilic degree that is average of frc and spr terms in this table; [d]The values of MET CG can be updated as 0.11 for spr, 0.19 for frc, and 0.15 for avg if we consider additional statistics of h_{elec} that we did not include in calculating this table.
